# Microbiota Alterations in Precancerous Colon Lesions: A Systematic Review

**DOI:** 10.3390/cancers13123061

**Published:** 2021-06-19

**Authors:** Francesca Aprile, Giovanni Bruno, Rossella Palma, Maria Teresa Mascellino, Cristina Panetta, Giulia Scalese, Alessandra Oliva, Carola Severi, Stefano Pontone

**Affiliations:** 1 Department of Translational and Precision Medicine, Gastroenterology Unit, Sapienza University of Rome, 00161 Rome, Italy; francesca.aprile@uniroma1.it (F.A.); giovanni.bruno@uniroma1.it (G.B.); giulia.scalese@uniroma1.it (G.S.); carola.severi@uniroma1.it (C.S.); 2 Department of Surgical Sciences, Sapienza University of Rome, 00161 Rome, Italy; rossella.palma@uniroma1.it (R.P.); cristina.panetta@uniroma1.it (C.P.); 3 Department of Public Health and Infectious Diseases, Sapienza University of Rome, 00161 Rome, Italy; mariateresa.mascellino@uniroma1.it (M.T.M.); alessandra.oliva@uniroma1.it (A.O.)

**Keywords:** microbiota, adenoma, colorectal cancer, Proteobacteria, *Fusobacteria*, polyp, gut, *Bacteroides*, metabolomics, endoscopy

## Abstract

**Simple Summary:**

Even with recent advances, gut microbiota is still one of the most demanding challenges that research needs to handle. In particular, given its deep impact on gastrointestinal health, microbiota could explain the development and progression of certain diseases. Moreover, it could be used as a potential predictive biomarker. Given this, the relationship between intestinal microbiota and colorectal adenoma, considered a premalignant lesion leading to carcinoma, has been deeply evaluated. This review highlights the historical and novel data on microbiota characteristics in adenoma patients to provide an updated summary of current knowledge and its limits.

**Abstract:**

Gut microbiota plays an important role in human health. It may promote carcinogenesis and is related to several diseases of the gastrointestinal tract. This study of microbial dysbiosis in the etiology of colorectal adenoma aimed to investigate the possible causative role of microbiota in the adenoma–carcinoma sequence and its possible preventive role. A systematic, PRISMA-guided review was performed. The PubMed database was searched using “adenoma microbiota” and selecting original articles between January 2010 and May 2020 independently screened. A higher prevalence of Proteobacteria, Fusobacteria, and Bacteroidetes phyla was observed in the fecal luminal and mucosa-associated microbiota of patients with adenoma. However, other studies provided evidence of depletion of *Clostridium*, *Faecalibacterium*, *Bacteroides* and *Romboutsia*. Results on the relationship between adenoma endoscopic resection and microbiota were inconsistent. In conclusion, none of the analyzed studies developed a predictive model that could differentiate adenoma from non-adenoma patients, and therefore, to prevent cancer progression. The impact of adenoma’s endoscopic resection on microbiota was investigated, but the results were inconclusive. Further research in the field is required.

## 1. Introduction

Colorectal cancer (CRC) is the third most prevalent cancer and the second-leading cause of cancer-related deaths worldwide. In 2020, more than 1.9 million new cases of colorectal cancer (including anus) and 935,000 deaths were estimated. In transitioned countries, the decline in colorectal cancer incidence is linked to adoption of colonoscopy screening programs and heathier lifestyle [1]. In this setting, it is important to understand the mechanisms causing this disease and the possible markers that could predict it to prevent the possible development of CRC. Fearon and Vogelstein first described the role of genetic alterations in the progression from adenomas to CRC, a process known as the *adenoma–carcinoma sequence* [2]. Thus, early detection of adenomas and their endoscopic removal are crucial to avoid the development of CRC. Although specific etiologic agents responsible for adenomas are not fully known, several genetic and environmental risk factors have been involved, in particular, the Western diet and the gut microbiota. Accordingly, the hypothesis that bacterial pathogens might play a key role has gained more and more attention [3]. In fact, the microbiota causes mucosal permeability alterations, bacterial translocation, and activation of the immune system, leading to chronic inflammation [4]. Therefore, a comprehensive characterization of the microbiota in patients with adenomas is of great importance to define its potential as a diagnostic marker of epithelial overgrowth and as a prevention tool. In this review, we discuss the characteristics of the microbiota in patients with adenomas and their implications, along with possible future applications.

## 2. Material and Methods

According to the PRISMA guidelines, we performed a systematic review to investigate the characteristics of the microbiota in adenoma patients. The online database PubMed (up to 5 May 2020) was searched using the following term: “Adenoma microbiota” and selecting original relevant articles published between January 2010 and May 2020. The research and selection of studies was independently performed by R.P. and C.P. All bibliographic references of the selected studies were evaluated for any relevant studies not found by the search platform. Reviewers resolved disagreements by discussion, and unresolved issues were resolved by a third reviewer (G.B.). For the purpose of our review, the inclusion criteria were as follows: Analyzing gut microbiota in human fecal samples or intestinal mucosa in adenoma cases. Exclusion criteria were studies conducted in mice, studies that analyzed microbiota, editorial publication type, and reviews. The primary outcome was identifying possible differences in the constitution of microbiota in patients with or without colonic adenoma, analyzing the role of the different bacterial phyla in colorectal adenoma genesis.

## 3. Results

**Figure 1:**
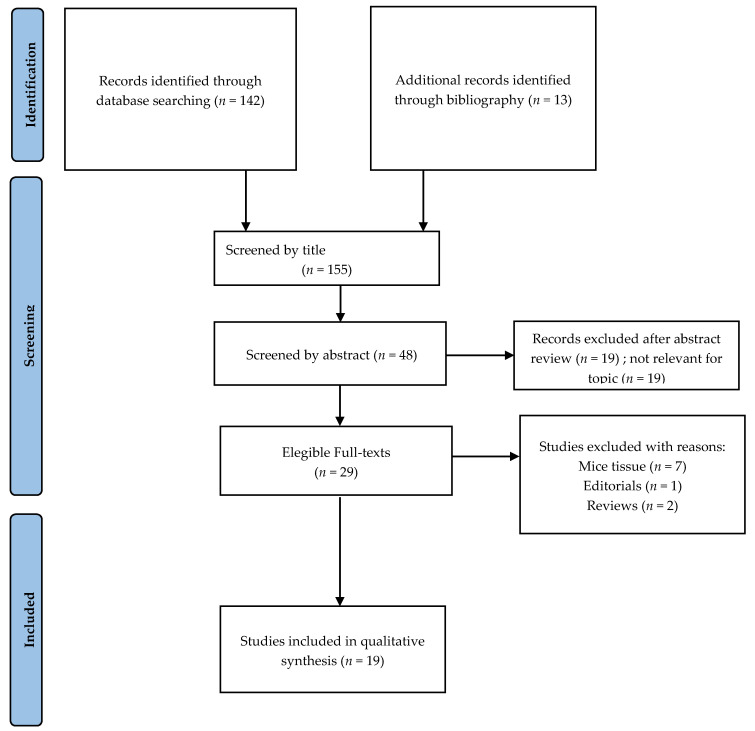
Literature search flowchart diagram for systematic review.

Our search returned 155 articles in PubMed, of which we excluded 107 after reading the abstracts (Figure 1). Nineteen full-text articles published in the English language met the reviewers’ inclusion criteria, and were, therefore, included. Of the 19 studies included, ten analyzed gut microbiota from stool samples, while nine analyzed gut microbiota from the intestinal mucosa. However, stool partially represents the overall autochthonous bacterial communities that are in direct contact with the intestinal mucosa, so we included those studies.

Amongst the selected studies, three used qPCR for quantitative real-time amplifica-tion of bacterial DNA [5,6,7] and expressed the results as cycle thresh-old (CT), Log Transformed Copy Number and 2^−ΔΔCt^, respectively; two studies [8,9] used metagenomic sequencing; 13 studies used 16S rRNA sequencing and the remaining study [10] used ENTERO-test 24 plus MALDI-TOF mass spectrometry for bacterial identification. Three additional studies [11,12,13] also used qPCR in addition to 16S rRNA gene sequencing. Details of the 19 included studies are shown in Table 1.

## 4. Discussion

### 4.1. Adenomas

Precancerous polyps are broadly categorized as traditional adenomas and serrated polyps. These different forms involve two different pathways. Traditional adenomas use the so-called “conventional pathway.” The alteration of the Wnt-β-catenin pathway is related to some allelic losses at the level of the adenomatous polyposis (APC) gene. Serrated polyps include hyperplastic polyps (75%), which do not progress into dysplastic forms; traditional serrated adenomas (TSAs) (5%); protruded lesions (sessile or pedunculated) lesions compatible with conventional adenomas (CA); and sessile serrated adenomas (SSAs). In SSAs, crypt proliferation leads to asymmetric structures; they may also have a yellow mucous cap, making endoscopic identification easier. The progression from serrated adenomas to CRC goes through the “serrated pathway,” which manifests itself through an accumulation of epithelial cells, caused by altered cell migration or apoptosis. Considering the molecular way, the BRAF mutation is more frequent in SSAs [14], which is rarely present in traditional adenomas; this supports the concept that the “serrated pathway” represents an alternative route to CRC [15].

In clinical settings, the number and structure (shape, size, and type) of adenomatous polyps are reliable tools for predicting which patients are more prone to develop CRC based on polyp morphology. Recent European Society of Gastrointestinal Endoscopy (ESGE) guidelines recommend the correct timing of post-polypectomy surveillance colonoscopy, considering all endoscopic, histological, and patient-related factors [16].

### 4.2. Microbiota

The human microbiota comprises 10–100 trillion cells, thousands of species, and at least 20 million unique microbial genes that participate actively in the host’s health. Several studies have focused on intestinal microbiota interactions and their characteristics. These studies highlighted that the intestinal microbiota plays a major role in the host immune system by supplying energy for host cell metabolism, in food digestion, nutrient absorption, fermentation of dietary fiber, and metabolism of xenobiotics [17,18,19,20]. It also contributes to regulating cell proliferation, differentiation, and gene expression in host epithelial cells [21]. Intestinal microbiome is also involved in the pathogenesis of CRC, as it works as an environmental modifier through its influence on gut homeostasis and intestinal immune pathways. In particular, a recent meta-analysis revealed a core set of 29 species to be enriched in CCR metagenomes [20,21]. More than ten different phyla were found to contribute to the balance of the gastrointestinal (GI) tract, with a prevalence of Firmicutes and Bacteroidetes [22]. Microbiota studies use α-diversity and β-diversity as valuable measures to define microbiota in patients with adenoma compared to non-adenoma patients. The former is the variation of microbes in a single sample expressed by richness and evenness. By contrast, β-diversity indicates the variation in microbial communities between samples in terms of ecological distance.

The intestinal microbiota resides in two different areas: Luminal microbiota (LM) and mucosa-associated microbiota (MAM). The LM occupies the intestinal lumen and its sampling is easily obtained by collecting stool. Therefore, most large-scale studies on gut microbiota have analyzed fecal luminal microbiota [22,23]. By its nature, LM is most affected by dietary changes. On the other hand, MAM, which resides on the epithelial surface, has a greater interaction with host cells than LM [4,24]. Thus, MAM plays a more relevant role in the adenoma–carcinoma sequence because of its close contact with the intestinal and host immune cells.

**Figure 2:**
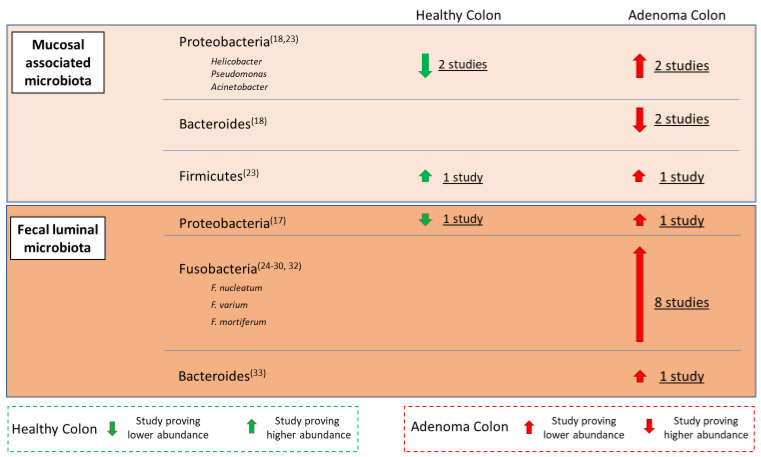
Distribution of main bacterial families of human microbiota in the physiological condition and in adenoma patients (the thickness of the arrows is linked to the strength of the scientific evidence drawn from the literature).

Nevertheless, endoscopic biopsies are required during a colonoscopy, which is an invasive procedure. Moreover, bowel preparation for colonoscopy could cause changes in MAM [25]. These bacterial communities may relate differently to gastrointestinal diseases; therefore, several trials investigated the possible role of intestinal dysbiosis in the CRA pathogenesis (Figure 2).

### 4.3. Proteobacteria

Higher bacterial diversity and richness have been shown in fecal specimens of patients with colonic polyps with a significant abundance of Proteobacteria. The predominance of these gut pathogens in colorectal adenoma (CRA) patients may differentiate them from healthy subjects. Sophisticated statistical methods, such as rank-based distance metrics and random forests plus leave-one-out, were performed to quantify and test differences in composition (β-diversity) and discrimination between CRA and controls [26]. A study on adherent microbiota hypothesized that bacterial composition obtained from biopsies on normal rectal mucosa may reflect the presence of adenoma bacterial communities. They observed that gut adherent microbiota differed significantly in subjects with adenomas from that of control subjects without adenomas and confirmed an higher proportion of Proteobacteria together with a lower amount of *Bacteroidetes* between CRAs and control patients [27,28]. Later, the association between the gut microbiota and the presence of adenomas anywhere in the colon, found through collecting biopsies from normal rectal mucosa, was evaluated through the more sophisticated method of pyrosequencing of 16S rRNA tags from genomic DNAs. Again, the authors found higher microbial richness in 33 adenoma subjects compared to 38 adenoma-free controls. In particular, *Pseudomonas*, *Helicobacter*, *Acinetobacter*, and other genera belonging to the phylum Proteobacteria were found in higher relative abundance in cases compared to controls [13]. By altering the gut environment, these pathogens may potentially increase the risk of adenoma through microbe-specific patterns. Accordingly, possible mechanisms to explain the association between microbes and adenoma development were hypothesized. *Helicobacter* alters the pH of the gastrointestinal tract, while *Acidovorax* spp. is a Gram-negative *Proteobacter* that potentially induces local inflammation by increasing flagellar proteins. Moreover, considering the relationships between CRC and obesity and the previously described relationship between obesity and microbiome [29,30], the authors investigated the association of gut microbiota with BMI and waist–hip ratio (WHR) in obese patients with or without colon adenoma. However, no significant differences in microbial richness and evenness were correlated to BMI in patients with or without adenoma, suggesting that microbial community membership and structure could have a higher impact on adenoma formation than on environmental factors, such as obesity [13].

In analyzing the studies conducted in Europe, an abundance of Proteobacteria was proven in the Slovak study, which included only ten patients with colorectal adenoma, ten with carcinoma, and nine healthy controls. As opposed to other studies, biopsy specimens were taken from normal mucosa, and both adenomatous and tumorous tissue [10]. By ENTERO-test 24 and MALDI-TOF mass spectrometry, significant differences were found in the presence of intracellular *E. coli* [10].

To better understand the link between bacterial microbiota and adenomatous tissue, other studies simultaneously investigated microbiota on healthy mucosa and on polyp samples. In particular, adenoma biopsy samples were compared to adjacent non-adenoma samples from the same patient. Interestingly, both tissues showed Firmicutes as the most predominant phylum, contributing about 50%, followed by Proteobacteria and Bacteroidetes, without remarkable differences in abundance across any of the sampled tissues in the same patient with adenoma. However, the profile of the intestinal mucosa-associated microbiota in 31 subjects with adenomas (adenoma biopsy samples and adjacent normal tissue samples) showed significantly higher bacterial diversity and richness than in 20 healthy controls. In particular, a relative expansion of Proteobacteria was observed in patients with adenomas, with a concomitant Firmicutes reduction. These findings may suggest that the driver–passenger hypothesis, in which “drivers” remodel the colonic bacterial community through colonic epithelial cell changes and then disappear from cancerous tissue to be replaced by passenger bacteria, may not be fully relevant to the precancerous colon lesion [31].

### 4.4. Fusobacteria

Other studies revealed that *Fusobacteria* is prevalent in premalignant colorectal lesions [5,6,8,9,28,32,33,34,35,36]. In particular, *Fusobacterium nucleatum* was found in abundance in the stools of 50 patients with adenomatous polyps (AP), mainly consisting of villous polyps/tubular villous polyps (TVP), in contrast to samples from the normal, hyperplastic polyps (HP) and SSA groups. The prevalence of *F. nucletaum* in AP was associated with a higher abundance of bacteria belonging to other phyla (*E. faecalis*, *S. bovis*, *Bacteroides fragilis (ETBF)*, and *Porphyromonas* spp.) and a lower number of *Lactobacillus* spp., *Roseburia* spp., and *Bifidobacterium* spp., compared to the healthy controls, HP and SSA. This evidence suggests that gut bacterial quantity correlates with the size, location, and grade of dysplasia of polyp cases and, thus, that gut microbiota might contribute to early stages of colorectal carcinogenesis through the development of AP, but not SSA. Unfortunately, these observed microbial alterations lack the sensitivity and specificity to serve as clinical biomarkers for adenoma detection [5].

The relative abundance of *Fusobacterium nucleatum* in adenomas was confirmed in another study independently from the histopathology of premalignant colorectal lesions (serrated and non-serrated adenomas), even if F. nucleatum was lower in adenomas than in CRC [6].

Furthermore, both the development of CRA and intramucosal CRC have been associated with *Fusobacterium varium*. The metagenomic study of fecal aspirates during colonoscopy in CRA, CRC and healthy subjects, has shown, thanks to the use of next-generation sequencing (NGS), a prevalent presence of *Fusobacteriales* and *Fusobacterium* in CRA patients in contrast to healthy subjects. Between these, *F. varium* is present in about 80% of CRA patients and takes on a distinctive value between these subjects and healthy subjects. However, the regional limitation of this study, performed on the Japanese population, could profoundly impact the results that reveal a prevalent presence of *F. varium* instead of the more common *F. nucleatum*. These variations suggest region-to-region discrepancies regarding the colonization of *Fusobacterium* spp. and a possible, but not yet sufficiently studied relationship between microbiota and human ethnic groups [32].

*Fusobacterium mortiferum*, another relevant species of the Fusobacteria phylum, was significantly increased in a small court of 35 patients with pathologically diagnosed adenomatous polyps and was associated with a significant decrease in *Faecalibacterium prausnitzii* and *Bifidobacterium pseudocatenulatum* [8]. The microbiome cluster characterized by *Faecalibacterium prausnitzii* was shown to be positively correlated with the fatty acid biosynthesis pathway of butyrate, which is known to protect the host from CRC development [8,28,32,33,34] and probably suppress chronic intestinal inflammation by regulating T cells [35].

The association of Fusobacteria with premalignant lesions has been further substantiated by studies on resident microbiota [13]. Using methods such as quantitative PCR of the 16S ribosomal RNA gene and local cytokine gene expression (IL-6, IL-10, IL-12, IL-17 and TNF-α) the bacterial level related to the mucosal biopsies performed on the rectum was determined. Compared to controls, adenoma patients showed a significant increase in *Fusobacterium species.* Just the abundance of *Fusobacterium* in colonic mucosa and their correlation with tissue cytokines, could validate a strong link between the presence of adenomas and mucosal inflammation. However, no significant correlation was found between *Fusobacterium species* and adenoma size or number. This could be more weighty if we consider that there are no noticeable significant differences between adenoma and non-adenoma patients about other risk factors, such as alcohol and caloric intake, waist–hip ratio, body mass index, and total fat intake [36].

### 4.5. Bacterioides

A significant increase in *Bacteroides massiliensis* and *Bacteroides dorei* from healthy to advanced adenoma was observed by evaluating 156 metagenomic shotgun-sequenced fecal samples. *B. dorei* correlated with levels of C-reactive protein (CRP), a marker for acute inflammation, suggesting a potential role in adenoma formation [9]. The authors also studied the varieties and uniformity of the gut microbiota in healthy controls compared to patients with advanced adenoma or carcinoma. A progressive increase in the genetic variety was highlighted, with a progressive increase starting from the controls to reach the top in the carcinoma patients. Thus, in patients with advanced colorectal adenoma or carcinoma, greater richness in genes or genera likely indicates an overgrowth of a variety of harmful bacteria or archaea. Moreover, the authors considered patients’ diets (their consumption of fruits, vegetables, or red meat) to evaluate their potential influence on gut microbiota. Standardized questionnaires were administered to calculate the amount of one serving, type, frequency, relative fiber content (consumption of vegetables and fruits), and amount of meat and fish. High intake of red meat relative to fruits and impacts the outgrowth of bacteria, contributing to a more hostile gut environment and the colorectal adenoma–carcinoma sequence [9].

#### 4.5.1. Depletion of Bacterial Communities in Adenoma

Other studies revealed relative depletion of some species or the whole network in patients with adenoma. Chen et al. compared CRA microbial communities to healthy controls by high-throughput 454 pyrosequencing of fecal samples performed in 47 sex- and age-matched individuals with similar lifestyles, reducing confounding factors potentially affecting the composition of intestinal microbiota. Firmicutes spp., which accounted for 68.3% and 67.9% of bacterial communities in the healthy control (HC) and CRA group respectively, were the most represented, followed by Bacteroidetes. The CRA group showed distinct differences in the constitution of the fecal microbiota community compared to the HC group. Thus, while *Clostridium*, *Roseburia* and *Eubacterium* spp., together with the genera related to the fermentation of butyrate were less represented, on the other hand, *Enterococcus*, *Streptococcus* spp. and Proteobacteria phylum were preponderant in comparison to the HC group [12].

Studies have evaluated the impact of dietary fiber intake on the gut microbiota structure in adenoma patients. The energy and carbon balance of the colon is directed by the carbohydrate fermentation activity of the human intestinal microbiota. Short-chain fatty acids (SCFA), derived from undigested dietary carbohydrates, are indispensable for the host and for microbial cross-feeding communities. On the other hand, butyrate and propionate have the role of regulators of intestinal physiology and the immune response, as well as acetate constitutes the substrate for lipogenesis and gluconeogenesis [22]. There is therefore evidence that a lower dietary intake of fiber and a low production of SCFA is present in patients with advanced colorectal adenoma. Likewise, producers of butyrate and their product were more present in both HC and CRA subjects with a high fiber content [12]. Further studies have evaluated the gut microbiota with respect to different polyp histology and locations. In a large cohort of patients, Peters et al., for the first time, observed relevant differences between CA and hyperplastic polyps or SSAs, suggesting that gut bacteria may play distinct roles in the development of polyps with different location and histology [37]. CA cases had lower fecal microbial species richness, particularly in advanced CA cases. In relation to overall microbiota composition, only distal or advanced CA cases differed significantly from controls, with an evident pauperization of the operative taxonomic units of *Clostridia* (*Ruminococcaceae*, *Clostridiaceae* and *Lachnospiraceae*) and an enrichment in the Bacilli and Gammaproteobacteria classes, Enterobacteriales order and *Acti-nomyces* and *Streptococcus* genera [37].

The same conclusion was reached in a pilot study of 12 patients with colon polyps (four adenomatous and eight hyperplastic polyps) performed on biopsies from colonic mucosa with polyp (CMP) and healthy marginal tissue (HMT). Firmicutes was the dominant phylum of the colonic mucosa samples, outnumbering the Bacteroidetes and Proteobacteria phyla. Interestingly, members of the Actinobacteria phylum, such as the *Bifidobacterium* genus, were relatively more abundant in the HMT than in the CMP samples. Instead, *Faecalibacterium*, *Bacteroides*, and *Romboutsia* were shown to be depleted in adenomatous polyps. In particular, *Romboutsia* may play a key role in maintaining the health status of the host, and it is suggested as a possible biomarker of intestinal dysbiosis [11].

#### 4.5.2. Controversial Results on the Role of Microbiota in Adenoma Development

The results of some research on adenoma have suggested no role of microbiota in the adenoma–carcinoma sequence. A study from Korea enrolled a total of 24 subjects: Three subjects of each gender for healthy patients, patients with CA, SSA, and CRC. Although in the absence of statistically significant differences in the MAM composition, this study confirmed that the presence of Proteobacteria is greater in MAM than in LM. However, the proportion of Proteobacteria in all groups analyzed was excessive compared to the literature (Proteobacteria [55.6%], Firmicutes [27.4%], Bacteroidetes [11.6%], *Fusobacteria* [3.2%], and *Actinobacteria* [1.7%]), with the suspicion of Proteobacteria contamination in an ecosystem where they are ubiquitous [38].

In addition, another study suggested no significant role of mucosa-associated gut microbiota in colorectal carcinogenesis. This study evaluated the abundance of *Fusobacteria* in tubular adenomas and in SSAs without finding any difference between them. Higher prevalence was observed in the CRC group (33.8%) than in the adenoma groups. However, the results were not compared to healthy controls, and the sample size was small (26 patients) [39].

#### 4.5.3. Metabolomics

Other studies went further, evaluating not only bacteria but also associated metabolites. A Chapel Hill group was the first to directly relate mucosal metabolites with bacteria in the development of colorectal adenomas and cancer. The metabolome in the normal rectal mucosal biopsies of 15 subjects with colorectal adenomas and 15 non-adenoma controls was assessed by liquid chromatography and gas chromatography time-of-flight mass spectrometry. Of the detected metabolites, 23 were found to have significantly lower concentrations in adenoma cases than in non-adenoma controls. Notably, in adenoma cases, they showed a decrease in the antioxidant-related metabolites 5-oxoproline and diketogulonic acid and an increase in the inflammatory metabolite prostaglandin E2. Moreover, they assessed the relationship between the metabolome and specific bacteria taxa and proved that it differed depending on adenoma status. Interestingly, significant increases in the abundance of *Bifidobacterium* sp. and *Eubacteria* were observed in colorectal adenomas. *Bifidobacterium* is a genus of lactic acid-producing bacteria that is generally viewed as probiotic; however, the relationship between *Bifidobacterium* sp. and CRC has been inconclusive [7]. A new study on gut metabolome analyzed the metabolomic profiles of patients with CA and CCR together with paired microbiota composition profiles. The association of a particular signature of microbiota in patients with adenoma and CCR was identified. The study revealed consistent changes in the carcinoma group of 24 out of 25 metabolites that were differentially abundant in the adenoma group compared to the HC. However, in carcinoma patients most of metabolite-level seemed to be weaker than in CA. Moreover, associations between the microbiome and metabolome were found and signatures of adenoma were defined as pairs of bacterial taxa and metabolomic features. It highlights the potential role of metabolites in predicting colorectal adenoma and potentially prevents future CRC [40].

#### 4.5.4. Microbiota and Adenoma Resection

Microbial alteration after CRA resection and its role in CRA recurrence has also been investigated, although by only a few studies. This field of research has the potential for high impact because the postoperative fecal microbiota may assume a role as a biomarker in the prediction of risk, timing of follow-up, and potential prevention. In one study, 67 individuals were evaluated before and after treatment for adenoma (*N* = 22), advanced adenoma (*N* = 19), and carcinoma (*N* = 26) by sequencing the 16S rRNA genes in stool samples [40]. In carcinoma patients, a pre- and post-treatment difference was observed, whereas, in adenoma and advanced adenoma, a shift toward a normal colon community was not experienced after treatment. It is likely that since the higher inflammatory environment in carcinoma has a greater impact on the structure of the microbial community, the effect of carcinoma therapy and its removal could have a greater effect on microbiota than adenoma endoscopic resection [41].

However, different results were reached in a study that aimed to investigate how new endoscopic treatments could impact the gut bacteria environment. A prospective study was conducted on 20 patients who underwent endoscopic resection of colorectal adenoma. The study examined the microbiota before and three months after adenoma resection. After adenoma resection, the overall microbial composition was slightly affected, and only *Parabacteroides* significantly increased post-operatively (3.8% vs. 1.5%). Nevertheless, a microbiota signature of *Parabacteroides*, *Streptococcus*, and Ruminococcus was constructed with the optimal discriminating performance of the post-operative status, with an accuracy of 78.8%. Even if further validation is needed in a larger sample size, the authors speculated that the persistent existence of microbial dysbiosis might suggest potential adenoma recurrence [42].

## 5. Conclusions

Growing evidence suggests microbial dysbiosis is a crucial environmental factor in the initiation of precancerous lesions of CRC. In particular, adenomatous tissue features an increased abundance of Proteobacteria and *Fusobacteria*. However, none of the suggested models was externally validated to differentiate between carriers of adenomas and healthy controls, and none of the included studies firmly assessed a definite biomarker that could be used to establish recurrence after treatment or to suspect adenomatous lesions.

Several limitations should be considered in interpreting the results on gut microbiota: Sample size is usually small, and samples are different. Moreover, the majority of the data comes from North America and Europe, with only a few studies from Africa or South America; this may indicate bias because environmental factors (diet and lifestyle) impacts gut microbiota. Thus, although gut pathogens and intestinal mucosa have been proven to be linked, further studies are needed to assess the molecular mechanism and definite clinical use of microbiota in the prediction of disease.

## Figures and Tables

**Table 1:** Details of studies (*n* = 19) included in the review. CRA: colorectal adenoma; CRC: colorectal carcinoma.

Reference	Year	Methods for Microbiota Analysis	qPCR	N. of Patients	Samples	Area
Liang et al.	2020	Metagenomic sequencing	NA	35 (adenomatous polyps)	Stool	China
Yu et al.	2019	16S rRNA gene sequencing	NA	20 (CRA)	Stool	China
Mangifesta et al.	2018	16S rRNA gene sequencing; qPCR (*Fusobacterium nucleatum*).	The deduced cell number was evaluated by comparing the cycle threshold (Ct) values obtained with those from a standard curve. Standard curves were calculated from serial dilutions of a culture with a known cell number (as determined by viable count assessment) for the bacterial strain versus Ct produced for each target gene. Results are expressed as genome copy numbers/gr.	12 (4 adenomatous polyps, 8 hyperplastic polyps)	Biopsy of Colonic Mucosa with Polyp and Healthy Marginal Tissue	Italy
Rezasoltani et al.	2018	Absolute qPCR (*Streptococcus bovis/gallolyticus*, *Enterococcus faecalis*, Enterotoxigenic *Bacteroides fragilis*, *F. nucleatum*, *Porphyromonas* spp., *Lactobacillus* spp., *Roseburia* spp. and *Bifidobacterium* spp)	The standard curve was plotted, by eight dilution points each tested in duplicate, using DNA obtained from reference strains. Results are expressed as CT.	118 (31 normal controls, 21 hyperplastic polyp, 16 sessile serrated polyp, 29 tubular adenoma, 21 villous/tubuvillous polyp)	Stool	Iran
Wachsmannova et al.	2018	ENTEROtest 24 plus MALDI-TOF mass spectrometry Gentamicin-protection assay to distinguish intracellular bacteria	NA	29 (10 CRA, 10 CRC, 9 healthy subjects)	Biopsy samples	Slovakia
Sze et al.	2017	16S rRNA gene sequencing	NA	67 (adenoma, *N* = 22, advanced adenoma, *N* = 19, carcinoma, *N* = 26).	Stool	USA
Yoon et al.	2017	16S rRNA gene 454-pyrosequencing	NA	24 (healthy control, conventional adenoma, sessile serrated adenoma, CRC, each *n* = 6)	Biopsy samples	Korea
Lu et al.	2016	16S rRNA gene pyrosequencing	NA	51 (31 adenoma, 20 healthy volunteers)	Adenoma mucosal biopsy samples and adjacent normal colonic mucosa	China
Park et al.	2016	16S rRNA gene pyrosequencing	NA	26 (8, tubular adenoma, 10 sessile serrated adenoma/polyp, 8 CRC)	Colorectal mucosal tissue	Korea
Goedert et al.	2015	16S rRNA gene sequencing	NA	61 (24 normal patients, 20 CRA, 2 CRC, 15 with other conditions)	Stool	USA, China
Nugent et al.	2014	qPCR (*Lactobacillus* sp., *Escherichia coli*, *Bifidobacterium* sp., *Clostridium* sp., *Bacteroide* sp., Eubacteria)	To generate a standard curve, the target 16S rRNA was amplified from a positive control strain by PCR. Results are expressed as Log Transformed Copy Number.	30 (15 adenoma, 15 adenoma-free control subjects)	Rectal mucosal biopsies	USA
Chen et al.	2013	16S rRNA gene 454-pyrosequencing	qPCR assays to determine the amounts of total bacteria, *Bacteroides* genus, and *Bifidobacteria* spp. A constructed plasmid was chosen to create a 10-log fold standard curve.	94 (47 sex- and age matched patients with advanced CRA and healthy subjects)	Stool	China
Sanapareddy et al.	2012	16S rRNA gene 454 titanium pyrosequencing	Abundance of a specific taxon was calculated by the delta–delta threshold cycle (DDCt) method.	71 (33 subjects with adenomas and 38 subjects without adenomas (controls)	Mucosal biopsies	USA
Shen et al.	2010	16S rRNA gene sequencing	NA	44 patients (normal colonic mucosa of 21 adenoma and 23 non-adenoma subjects)	Colorectal biopsies	USA
Feng et al.	2015	Metagenomic sequencing	NA	147 (57 healthy controls, 44 advanced adenoma, 46 carcinoma)	Stool	Austria
Ito et al.	2015	Quantitative PCR for *F. nucleatum.*	The Ct values for *F. nucleatum* were normalized to prostaglandin transporter (PGT) comparative analysis of the cycle thresholds (DCt). Results were expressed as 2^-ΔΔCt^	465 premalignant lesions (343 serrated lesions and 122 non-serrated adenomas) and 511 CRC	Tumor tissue specimens	Japan
Kim et al.	2020	16S rRNA gene sequencing	NA	240 (patients with advanced adenoma, *N* = 102), matched controls *n* = 102), patients with CRC, *N* = 36)	Stool	USA
Peters et al.	2016	16S rRNA gene sequencing	NA	540 Conventional adenoma cases (*N* = 144), serrated polyp cases (*N* = 73), or polyp-free controls (*N* = 323).	Stool	USA
Saito et al.	2019	16S rRNA gene sequencing	NA	81 (47 CRA, 24 intramucosal colorectal cancer, 10 healthy subjects)	Colonoscopy aspirates	Japan
